# 

**Published:** 1913-12-13

**Authors:** 


### THE MEMORIAL TO LORD LISTER.

Contributions recently made to the Lister Memorial Fund include the Newcastle-on-Tyne Committee, £46 7s.; members of the Newcastle-on-Tyne Clinical Society, £61 9s.; Newcastle-on-Tyne Clinical Society, £5 5s.; British Medical Association, Ceylon branch, £14 7s. 9d.; Burma branch, £19 3s. 7d.; Otago division, New Zealand branch, £5 5s.; Union of Swedish Hospital Surgeons, £20; University of Nancy (Faculty of Medicine), 100f.; University of Bordeaux (Faculty of Medicine), 150f.; Nottingham Medico-Chirurgical Society, £6 9s.; Plymouth Medical Society, £5 5s.; Wigan Medical Society, £2 2s. Donations may be sent to the honorary treasurers of the fund at the Royal Society, Burlington House, London, W.

Rates of Subscription: Twelvemonths, 6 6; Six months, 4/-; Three months, 2/-, post free to any address in the United Kingdom. Abroad, Twelvemonths, 8/8; Six months, 5/-; Three months, 2,6, post free.—Address The Subscription Dept., "The Hospital," The Hospital Building, 28/29 Southampton Street, Strand, London, W.C.

